# MR-guided focused ultrasound thalamotomy for lithium-induced tremor: a case report and literature review

**DOI:** 10.3389/fneur.2023.1331241

**Published:** 2024-02-01

**Authors:** Kate Gelman, Joseph Melott, Vishal Thakur, Abdul R. Tarabishy, Ana Brandt, Peter Konrad, Manish Ranjan, Adeel A. Memon

**Affiliations:** ^1^School of Medicine, West Virginia University, Morgantown, WV, United States; ^2^Department of Neurosurgery, Rockefeller Neuroscience Institute, West Virginia University, Morgantown, WV, United States; ^3^Department of Neuroradiology, Rockefeller Neuroscience Institute, West Virginia University, Morgantown, WV, United States; ^4^Department of Neurology, Rockefeller Neuroscience Institute, West Virginia University, Morgantown, WV, United States

**Keywords:** tremor, lithium, MR-guided focused ultrasound (MRgFUS), high-intensity focused ultrasound (HIFU), case report

## Abstract

Drug-induced tremor is a common side effect of lithium with an occurrence of approximately 25% of patients. Cessation of the offending drug can be difficult, and many medical treatments for drug-induced tremor are ineffective. Deep brain stimulation (DBS) has been shown in a limited number of case reports to effectively reduce drug-induced tremor, however, which remains an invasive therapeutic option. MR-guided focused ultrasound (MRgFUS) thalamotomy is an FDA-approved non-invasive treatment for essential tremor (ET). To the best of our knowledge, MRgFUS thalamotomy has never been reported to treat drug-induced tremor. Here, we present a case of a left-handed 55-year-old man with a progressive, medically refractory lithium-induced tremor of the bilateral upper extremities. The patient underwent MRgFUS thalamotomy targeting the right ventral intermediate nucleus (VIM) of the thalamus to treat the left hand. There was almost complete resolution of his left-hand tremor immediately following MRgFUS. There were no side effects. The patient continues to show excellent tremor control at 90-day follow-up and remains free from side effects. This case demonstrates MRgFUS thalamotomy as a possible novel treatment option to treat drug-induced tremor.

## 1 Introduction

Tremor is a common side effect of many medications and can significantly impact quality of life (QoL). Psychotropic drugs, including neuroleptics and mood stabilizers, are the most common offenders owing to their long-term use and subsequent dependence (1). In 2018, the prevalence of antipsychotic use was 1.6% among adults in the US (2). Antipsychotic use is increasing, with the number of people prescribed at least one antipsychotic rising from 5.0 million in 2013 to 6.1 million in 2018 in the US (3). Tardive tremors seen with neuroleptics use have been reported in 2.4% of cases (4). Bipolar disorders affect more than 1% of people worldwide, and lithium continues to be the mainstay treatment. Tremor is seen in approximately 25% to 27% of patients taking lithium (1, 4). Similar to most drug-induced tremors, lithium-induced tremor is generally symmetric and predominantly postural or intentional. It may emerge at any time during lithium treatment and can range in severity from a minor inconvenience to debilitating (5, 6). As the number of neuroleptics and mood stabilizer prescriptions increases, this potentially debilitating adverse effect will impact more people (7). More effective treatments for drug-induced tremor are much needed.

Drug-induced tremors are typically managed initially by reducing the dose or stopping the offending medication. In psychiatric conditions such as bipolar disorder, this is often not recommended given the disease severity and dearth of suitable alternatives. If the tremor persists or the offending drug cannot be altered, symptomatic treatment with tremor-reducing medications is initiated. Amerika et al. conducted a systematic review of the literature to identify and analyze treatments for drug-induced tremor (8). Their review found that β-blockers, most notably propranolol, have shown the most effectiveness. Unfortunately, there are several downsides to using drugs to control drug-induced tremor (8). In patients with neuropsychiatric conditions, certain tremor-reducing medications may be contraindicated. For example, tetrabenazine is contraindicated in patients with depressive symptoms (8). There is a need for alternative options for the treatment of drug-induced tremor.

Given the uncertainty of the diagnosis and poor understanding of the underlying pathology, surgical options for drug-induced tremor have been hesitantly tried. There are very few reports of deep brain stimulation (DBS) successfully attempted in these patients (Table 1) (8–11). However, DBS may be contraindicated in active or poorly controlled neuropsychiatric conditions, and certain DBS targets can aggravate mood and psychiatric symptoms when stimulated (12–14). Recently, MR-guided focused ultrasound (MRgFUS) thalamotomy emerged as a non-invasive effective treatment for ET and tremors of other etiologies (15, 16).

**Table 1:** Results from systematic review of the literature on surgical treatments for drug-induced tremor.

Study	Intervention and rationale	Drug	Tremor characteristics	Justification for drug-induced tremor diagnosis	Results
Rodrigues et al. (9) (United States)	DBS – VIM Left side As tremor predominantly affected QoL, DBS-VIM was chosen, as it is an established DBS target for treating Parkinsonian and ET	Haloperidol Aripiprazole	Bilateral UE rest, postural, and action tremor; intermittent head tremor Not responsive to propranolol, primidone, topiramate, clonazepam, gabapentin, or trihexyphenidyl Mild improvement with alcohol Mild Parkinsonism	Temporal relation between initiation of haloperidol and onset of hand tremor	Absence of R hand tremor at 12 months post-operatively
Milosevic et al. (10) (Canada)	DBS – Vop Right side Three trajectories were attempted intraoperatively (targeting the VIM, STN, and Vop). The trajectory targeting the Vop showed the most significant tremor suppression	Lithium	Bilateral rest and action/kinetic tremor Not responsive to alcohol or Propranolol No evidence of Parkinsonism	Temporal relation between starting Lithium and onset of tremor	Improvement of TETRAS score from 43 pre-operatively to 29 at 3 months post-operatively
Kashyap et al. (11) (United States)	DBS – STN Bilateral In addition to tremor, bradykinesia and rigidity also affected QoL, so the STN DBS target was chosen	Quetiapine Fluoxetine Trazodone	Bilateral UE and LE postural and kinetic tremor Not responsive to propranolol, amantadine, sinemet, pramipexole, or primidone Drug-induced Parkinsonism also present	Temporal relation to switching medications	Near-complete resolution of lower and upper extremity tremors at 2 years post-operatively
Amerika et al. (8) (United States)	DBS – VIM Bilateral As tremor predominantly affected QoL, DBS-VIM was chosen	Lithium	Progressive bilateral UE rest, postural, and kinetic tremor Not responsive to levodopa/carbidopa No evidence of Parkinsonism	Clinical presentation (progressive rest, postural, and kinetic tremor) and history of long-term use of lithium Temporary reduction of Lithium resulted in improvement of tremor	Improvement of TETRAS score from 28 pre-operatively to 4 at 3 years post-operatively

MRgFUS thalamotomy is commonly used to treat tremor via ablation of the ventral intermediate nucleus (VIM) of the thalamus (17). MRgFUS has been explored since the 1950s, but recent technological advances have allowed it to be used safely to target precise structures in the deep brain (18). In 2016, the FDA approved MRgFUS thalamotomy for essential tremor (ET) (15). It has been shown to reduce ET by 75%–89% (19, 20). MRgFUS thalamotomy has also been found to be effective for other non-ET tremors such as Parkinson’s disease (PD) tremor (15, 16). However, a comprehensive literature search of procedural treatment for drug-induced tremor yielded no published report of MRgFUS thalamotomy for drug-induced tremor.

**Figure 1:**
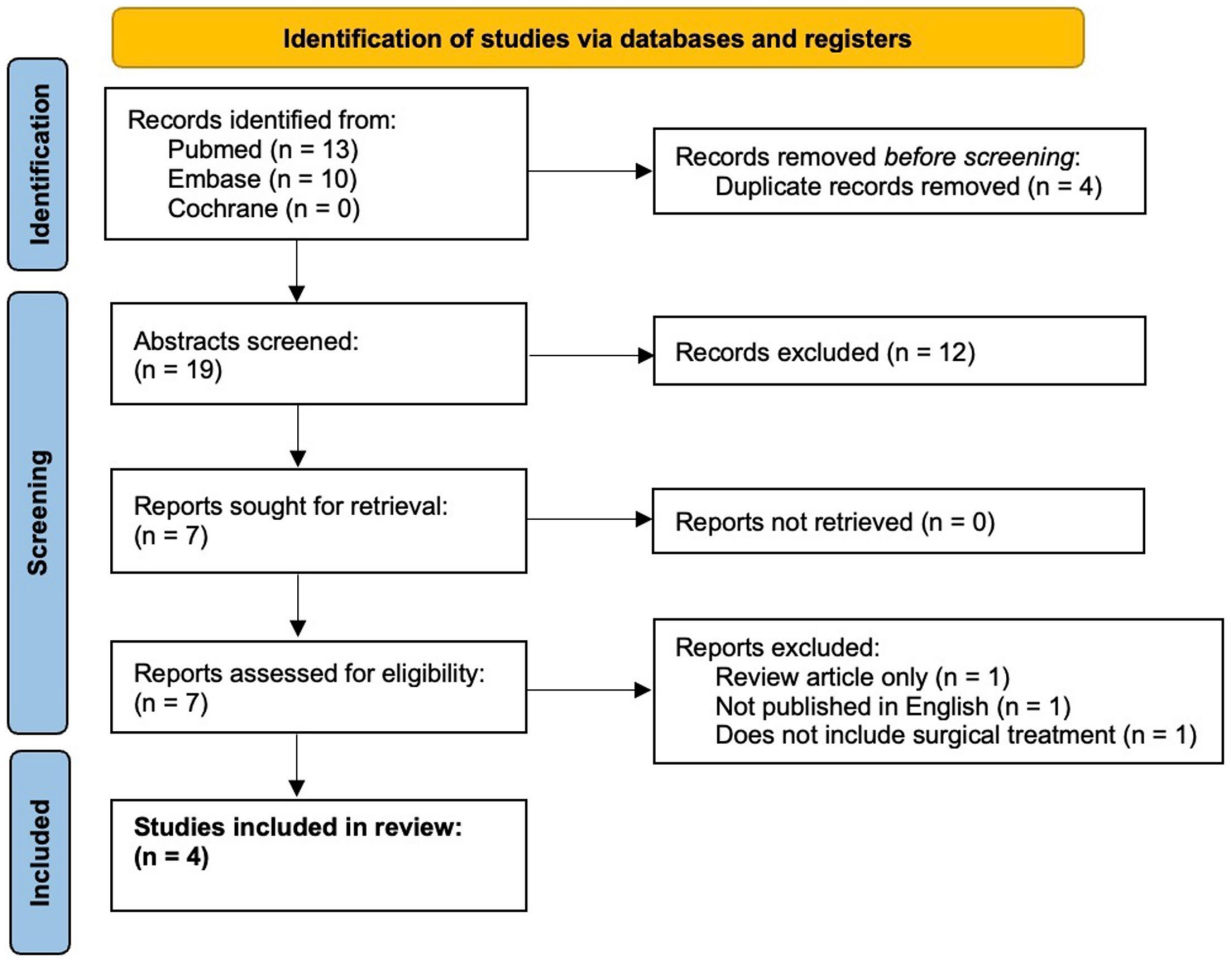
PRISMA flow diagram (21).

Here, we present a case of medically refractory lithium-induced tremor successfully treated with MRgFUS VIM thalamotomy. We also present a comprehensive literature review on surgical treatment of drug-induced tremor in PubMed, Embase, and Cochrane Libraries from January 1960 to 11 June 2023, following the Preferred Reporting Items for Systematic Reviews and Meta-Analysis guidelines (Figure 1) (21).

## 2 Case description

A 55-year-old left-handed man presented to the multidisciplinary movement disorder clinic for surgical evaluation of tremor. His past medical history was significant for bipolar 1 disorder. He had been treated with lithium for 20 years. He was also on bupropion, hydroxyzine, lamotrigine, metoprolol, omeprazole, primidone, propranolol, and ropinirole. Over the last 10 years, he developed a bilateral, progressive postural and action tremor of the upper extremities. He has no family history of ET or other movement disorders. No Parkinsonian symptoms were evident in him, such as bradykinesia, rigidity, shuffling in gait, or postural instability. The patient’s tremor significantly impacted his QoL. As an administrative officer, tremors impeded his fine motor skills, making it difficult to type, organize documents, and perform other office tasks. Due to challenges using utensils, potential food spills, and self-consciousness, tremors affected his psychosocial functioning outside the workplace. As a result of these challenges, he experienced personal and professional difficulties. He was unable to effectively perform his job and had significantly affected psychosocial function. Based on the presence of the temporal relationship with lithium and the absence of a strong family history of tremor, a predominantly high frequency and low amplitude postural and action tremors accompanied by jerky episodes that cause poor dexterity (22–24), he was diagnosed with drug-induced tremor.

When he presented to the multidisciplinary movement disorder clinic, he was taking a total of 120 mg of propranolol and 750 mg of primidone. His blood pressure was 98/66 during the clinic visit, his heart rate was 70, and he complained of being sluggish, so the propranolol dose was not recommended to be increased. We briefly discussed botulinum toxin injections with him. However, since he lives a 4-h drive away from the clinic, returning to the clinic every 3 months was not an option for him. Because his bipolar disorder had been well-controlled with lithium for 20 years, the 600 mg BID lithium dose was not altered. In addition, he could not recall whether the amount had ever been lowered to treat tremor in the past.

**Figure 2:**
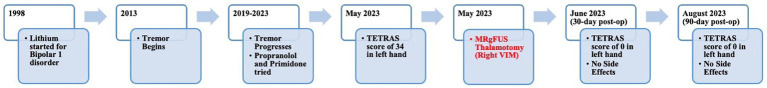
Timeline from initiation of lithium to the 90-day post-MRgFUS follow-up.

Institutional multidisciplinary movement disorder conference review approved surgical treatment for the refractory and disabling tremor. Given prior positive case reports, bilateral VIM DBS therapy was offered. However, the patient was apprehensive about receiving DBS surgery due to the invasiveness of surgery, the idea of having an implant, and the many follow-up appointments required as he lived 4 h from the clinic. A detailed discussion was held with him regarding MRgFUS irreversibility due to its ablative nature, unilateral treatment option at that time, and no case reports of this therapy being effectively used to treat drug-induced tremors. Despite these limitations, he decided to undergo MRgFUS right VIM thalamotomy for his left-hand tremor (Figure 2) since it is a minimally invasive procedure and requires fewer follow-up clinic visits.

## 3 Diagnostic assessment

Blood work revealed normal TSH and lithium levels. His score on the Activities of Daily Living Subscale of the Tremor Research Group Essential Tremor Rating Assessment Scale (TETRAS) was 34 (Supplementary Table S2). Physical examination revealed severe action and postural tremor and mild-to-moderate rest tremor (Supplementary Table S3; Supplementary Video 1). Based on clinical presentation and by ruling out other possible causes of tremor, the patient was diagnosed with medically refractory drug-induced tremor. As a part of the surgical evaluation, he underwent brain MRI and neuropsychological testing. Both were unremarkable.

## 4 Intervention

**Figure 3:**
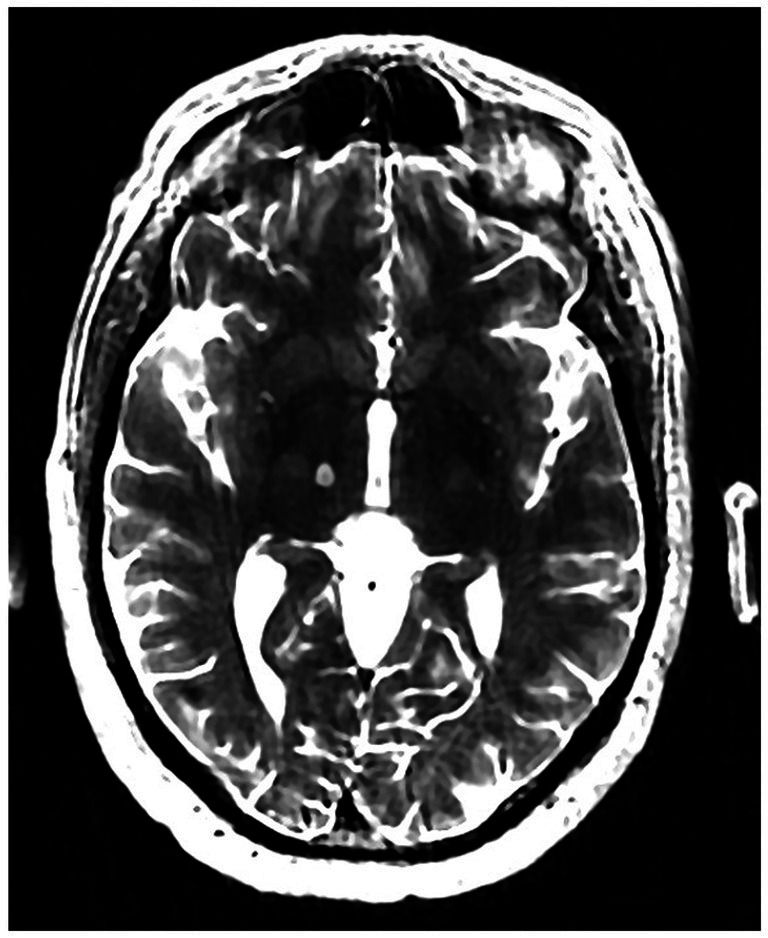
Volumetric T2-weighted image showing right VIM MRgFUS thalamotomy lesion.

The patient underwent MRgFUS thalamotomy targeting right VIM to treat the dominant left-hand tremor (Figure 3). The ablative temperature was reached with high-intensity focused ultrasound (HIFU) sonication with real-time MR guidance and clinical assessment. Tremor response and adverse effects were assessed between sonication.

**Figure 4:**
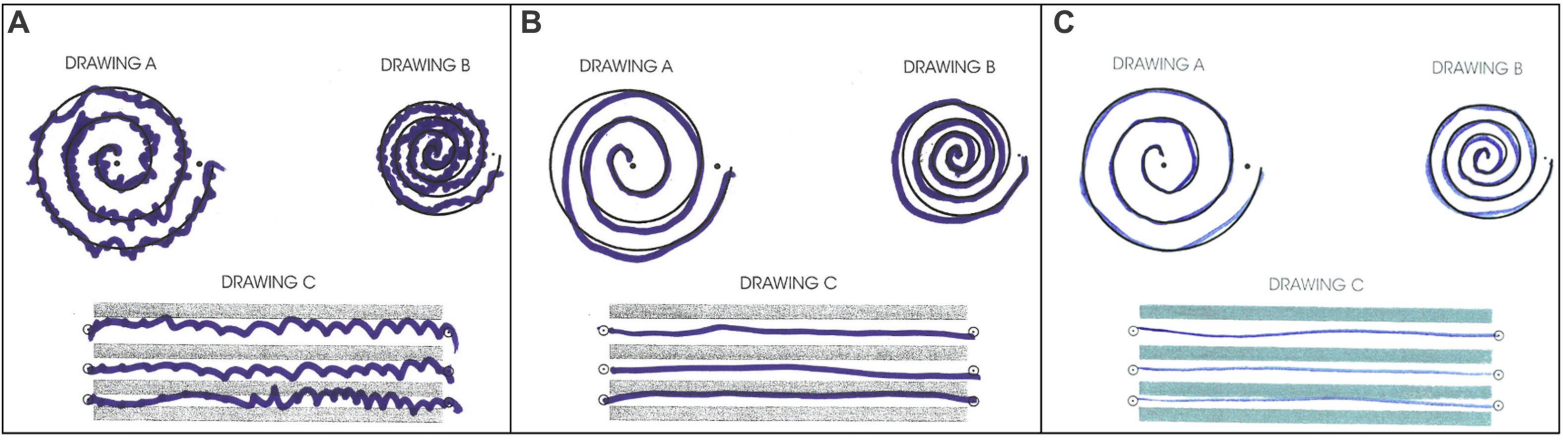
Archimedes spiral test during **(A)** pre-surgical evaluation, **(B)** immediate post-surgical examination, **(C)** 30-day follow-up post-surgical examination.

The patient tolerated the procedure well. Greater than 90% improvement of tremor was achieved during the procedure (Figure 4). There were no adverse effects including numbness, tingling, dysmetria, corticospinal tract abnormalities, dysarthria, gait abnormalities, or other neurological deficits. The patient was discharged on the same day after a few hours of observation.

### 4.1 Outcomes

The patient returned to his administrative office job and began normal socialization, including eating in restaurants, 2 weeks following the procedure, which was not possible prior to the procedure. At the 30-day and 90-day follow-up appointments, he showed no residual tremor or adverse effects (Figure 4). He reported “100% satisfaction.” His score on the TETRAS Activities of Daily Living Subscale was 0 for the left hand (Supplementary Table S2). Physical examination revealed no noticeable rest tremor and trace postural tremor and action tremor (Supplementary Table S3; Supplementary Video 3).

The patient enjoys singing bass in his church choir. Prior to the procedure, his embarrassment had prevented him from singing in public. At 30 days after the procedure, he reported elation that he had returned to singing with renewed confidence.

## 5 Discussion

Drug-induced tremor continues to be a challenge to providers and patients. It can cause constant impairment of simple activities, leading to significant decrease in QoL and aggravation of anxiety and depression symptoms associated with psychiatric condition. Taper or withdrawal of the offending medication is often not an option in cases of well-managed psychiatric disorders, and drug-induced tremor may persist even when withdrawal is achieved. Medications used to combat drug-induced tremor have conflicting support. While some medications have been shown to provide some benefit, most studies lack the necessary quality and sample size to suggest strong evidence for their use (8). A systematic literature review aimed at identifying procedural treatment options for drug-induced tremor revealed only four case reports, all of which reported the use of DBS. To the best of our knowledge, this is the first reported use of MRgFUS thalamotomy to treat refractory drug-induced tremor. This provides insight into an alternative, highly effective treatment option for a debilitating condition.

We conducted a systematic literature search from January 1960 to 11 June 2023 of surgical treatment for drug-induced tremor. The search yielded only four studies meeting full-text review criteria. All four studies were case reports reporting the use of DBS to successfully treat drug-induced tremor of the hand (8–11). The results varied from modest improvement to complete resolution of tremor (Table 1). Lithium was the culprit for the tremor in two studies (8, 10), and the VIM nucleus was the target in two studies (8, 9). There was no reemergence of tremor or worsening of psychiatric disorder in any of the cases across the follow-up periods ranging from 3 months to 3 years. These four cases demonstrate the effectiveness of DBS in drug-induced tremor; however, limitations should be noted. The small number of studies, and reliance on case reports may suggest publication bias and hinder generalization. Additionally, the unknown pathophysiological process and lack of definitive diagnostics or biomarkers in drug-induced tremor render uncertainty in diagnosis.

DBS for tremor has been widely used in PD and ET (15, 25). It is highly effective in reducing tremor in these disease states but carries risks and limitations across all applications (17). As an invasive procedure, it carries innate risks including infection (in approximately 4% of patients) and hemorrhage (in approximately 2.4% of patients) (26). In addition, several follow-ups are required for device programming. Additional surgeries may be needed to address hardware complications such as recurrent infections or malfunctions, and over time, there is a need for battery replacement if a non-rechargeable pulse generator is used (27). These factors could limit its acceptability by the patients already challenged with neuropsychiatric conditions. Moreover, poorly controlled psychiatric and mood symptoms are considered contraindications for DBS implantation (28). Excitingly, in 2016, unilateral MRgFUS thalamotomy was approved for the treatment of ET (15). MRgFUS is a non-invasive procedure that does not carry the risk of hardware-related complications seen with DBS, and it has been found to be as effective as DBS in the treatment of unilateral tremor (15). However, poor understanding of the pathophysiology of drug-induced tremor has prevented advances in its use as a surgical option for drug-induced tremor. Although there are recent reports of DBS for medically refractory drug-induced tremor, MRgFUS had never been tried. MRgFUS was preferred over DBS by our patient because it was surgically less invasive, involved fewer outpatient follow-up visits, and had no device complications despite irreversibility and experimentality of MRgFUS. The patient had immediate relief of tremor following MRgFUS thalamotomy, reducing his TETRAS score from 34 to 0 in his left upper extremity. At 30-day and 90-day follow-ups, TETRAS score was 0 for his left hand. To the best of our knowledge, this is the first case reporting successful MRgFUS VIM thalamotomy in drug-induced tremor, however, with only short-term follow-up.

MRgFUS could potentially be a more applicable tool in patients with psychiatric conditions where the use of DBS can be complicated. Recently, bilateral MRgFUS has been shown to be effective for ET if one side is done at a time and sufficient time elapses between procedures and was recently approved by the FDA (17, 29, 30).

Our study is not without limitations. This was an experimental treatment in a single case. The patient was diagnosed with drug-induced tremor based on the character of the tremor and the temporal relationship to lithium treatment. A diagnosis of drug-induced tremor is more reliable if the offending medication can be removed to expose a causal relationship. Unfortunately, this was not possible for our patient, whose psychiatric health is dependent on lithium. Furthermore, no neurophysiological studies were conducted, which would have enhanced diagnostic accuracy (24). Another limitation of the study is the possible presence of bradykinesia preoperatively. This raises the possibility of PD or drug-induced Parkinson’s disease, even though lithium rarely causes it. We believe that the slowness of hand movements was caused by the presence of tremor as previous studies have shown that tremor leads to prolonged reaction times due to incomplete muscle contractions, leading to a perception of bradykinesia (31, 32). Additionally, tremors can cause pacing effects as a result of voluntary movements (33, 34). Furthermore, slowness in left-hand movement was resolved after right VIM MRgFUS, supporting this hypothesis since VIM is not a target for bradykinesia. As a result of these factors, we concluded that the patient did not have Parkinsonism and thus we did not order a DaT scan. Although the short-term outcome in our case is excellent, the long-term outcomes are unknown and should be observed for any delayed side effects or re-occurrence of tremor. More cases need to be explored and followed longitudinally.

MRgFUS VIM thalamotomy offers a non-invasive, safe, and effective treatment option for medication induced tremor; however, larger studies with a longer follow-up are needed to validate the result.

## Data availability statement

The datasets presented in this article are not readily available because of ethical and privacy restrictions. Requests to access the datasets should be directed to the corresponding author.

## Ethics statement

The studies involving humans were approved by West Virginia University, School of Medicine, Department of Neurosurgery. The studies were conducted in accordance with the local legislation and institutional requirements. The participants provided their written informed consent to participate in this study. Written informed consent was obtained from the individual(s) for the publication of any potentially identifiable images or data included in this article.

## Author contributions

KG: Methodology, Writing – original draft, Writing – review & editing. JM: Methodology, Writing – review & editing, Writing – original draft. VT: Methodology, Writing – review & editing. AT: Visualization, Writing – review & editing. AB: Methodology, Writing – review & editing. PK: Methodology, Writing – review & editing. MR: Methodology, Writing – review & editing. AM: Conceptualization, Methodology, Supervision, Writing – review & editing.
