# Supplementary material for: MR-guided focused ultrasound thalamotomy for lithium-induced tremor: a case report and literature review
Source: Front Neurol. 2024 Feb 1;14:1331241. doi: 10.3389/fneur.2023.1331241 (PMC10867204; doi:10.3389/fneur.2023.1331241)
Supplement: Supplementary file 1 [file Data_Sheet_1.pdf]

Supplementary Material

1 Supplementary Figures and Tables

Table S1. Systematic Literature Review conducted June 11, 2023

**Search Strings**		
	**Search String**	**Number of Hits**
PubMed	("tardive tremor"[All Fields] OR "medication-induced tremor"[All Fields] OR "medication induced tremor"[All Fields] OR "drug-induced tremor"[All Fields] OR "drug induced tremor"[All Fields] OR “Lithium-induced tremor”[All Fields] OR “Lithium induced tremor”[All Fields] OR “Li induced tremor”[All Fields] OR “Li-induced tremor”[All Fields]) AND (surgery[MeSH Terms] OR "surgery"[All Fields] OR “surgical”[All Fields] OR "procedure"[All Fields] OR "procedure"[All Fields] OR "deep brain stimulation"[MeSH Terms] OR ("deep"[All Fields] AND "brain"[All Fields] AND "stimulation"[All Fields]) OR "deep brain stimulation"[All Fields] OR "DBS"[All Fields] OR “ablation"[All Fields] OR “HIFU"[All Fields] OR “High-Intensity Focused Ultrasound"[All Fields] OR “High Intensity Focused Ultrasound"[All Fields] OR “Focused Ultrasound"[All Fields] OR “GPi"[All Fields])	13
Embase	('tardive tremor' OR 'medication-induced tremor' OR 'drug-induced tremor' OR 'drug induced tremor') AND ('medical procedures' OR 'high frequency ultrasound' OR 'brain depth stimulation' OR 'focused ultrasound therapy' OR 'ablation therapy')	10
Cochrane Libraries	(("tardive tremor")OR ("medication-induced tremor") OR ("medication induced tremor") OR ("drug-induced tremor") OR ("drug induced tremor") OR (“Lithium-induced tremor”) OR (“Lithium induced tremor”) OR (“Li induced tremor”) OR (“Li-induced tremor”)) AND ([mh “surgery”] OR ("surgery") OR (“surgeries”) OR ("procedure") OR ("procedures") OR [mh “deep brain stimulation”] OR (("deep") AND ("brain") AND ("stimulation")) OR ((deep NEXT (brain*) NEXT (stimulation*))) OR ("DBS") OR (“ablation") OR (“HIFU") OR (“High-Intensity Focused Ultrasound") OR (“High Intensity Focused Ultrasound") OR (“Focused Ultrasound") OR (“GPi"))	0

Inclusion/Exclusion Criteria	
**Include**	**Exclude**
(1) peer-reviewed research studies, (2) included individuals diagnosed with drug-induced tremor, (3) studies specifically focused on surgical treatment as a therapeutic intervention for drug-induced tremor, (4) studies published in the English language	(1) wrong patient population; (2) studies with no outcome documentation; (3) review articles or commentaries only; (4) studies not published in the English language

Table S2. TETRAS Activities of Daily Living Subscale Scores.

Elble R, Comella C, Fahn S, Hallett M, Jankovic J, Juncos J, Louis E, Lyons K, Ondo W, Pahwa R, Sethi K. The essential tremor rating assessment scale (TETRAS). InMovement Disorders 2008 Jan 1 (Vol. 23, No. 1, pp. S357-S357). DIV JOHN WILEY & SONS INC, 111 RIVER ST, HOBOKEN, NJ 07030 USA: WILEY-LISS

Activity of Daily Living	Before	30 days after MRgFUS
1. Speaking	1	0
2. Feeding with a spoon	3	0
3. Drinking from a glass	3	0
4. Hygiene	2	0
5. Dressing	2	0
6. Pouring	3	0
7. Carrying food trays, plates, or similar items	4	0
8. Using Keys	3	0
9. Writing	3	0

10. Working	2	0
11. Most affected task: eating	4	0
12. Social impact	4	0
**TOTAL**	**34**	**0**

Table S3. Physical Examination Tremor Rating Scale Results

	Before	Immediately after MRgFUS	30 days after MRgFUS
Rest Tremor	2	0	0
Distal Postural Tremor	3	0.1	0.1
Proximal Postural Tremor	4	0.1	0.1
Action Tremor	4	0.25	0.25

1.1 Supplementary Figures

Supplementary Figure 1. Archimedes spiral test during **(A)** pre-surgical evaluation, **(B)** immediate post-surgical examination, **(C)** 30-day follow-up post-surgical examination

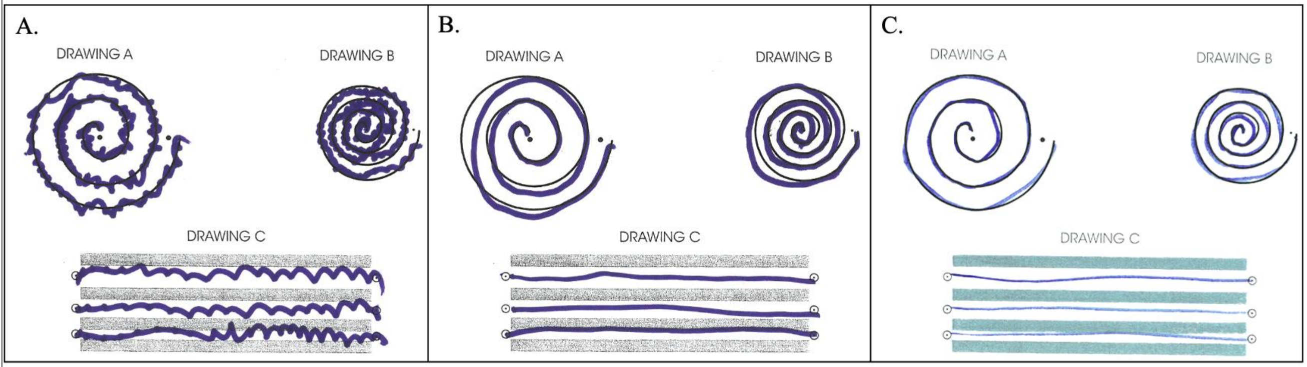

Supplementary Figure 1. Archimedes spiral test during **(A)** pre-surgical evaluation, **(B)** immediate post-surgical examination, **(C)** 30-day follow-up post-surgical examination
